# Three-dimensional alveolar bone assessment of mandibular molars for immediate implant placement: a virtual implant placement study

**DOI:** 10.1186/s12903-021-01849-w

**Published:** 2021-09-27

**Authors:** Haida Chen, Wei Wang, Xinhua Gu

**Affiliations:** Department of Stomatology, The First Affiliated Hospital, College of Medicine, Zhejiang University, #79 Qingchun Road, Hangzhou, 310003 Zhejiang Province People’s Republic of China

**Keywords:** Cone-beam computed tomography, Implants, Immediate placement, Mandible, Molar

## Abstract

**Background:**

To elucidate the anatomical features of the mandibular molar region to allow safe immediate implant placement.

**Methods:**

Cone-beam computed tomography images of 150 patients (600 teeth) were reviewed retrospectively. The virtual implants were placed in the mandibular first and second molar region. The anatomic structures of the mandible and inter-radicular septum were both categorized into three types. The relationship between implant and inferior alveolar nerve (IAN), and the horizontal distance from the implant surface to the bone wall were analyzed. Variables were compared using a student’s t-test, or Mann–Whitney U test.

**Results:**

Type U (39.0%) and type S (56.0%) were the most common in the first molar, while type U (67.7%) and type M (54.7%) had the highest prevalence rate in the second molar. The mean distance from the level where the virtual implant was completely surrounded by bone to IAN was 7.06 mm. The mean horizontal widths from the implant to the mesial and distal socket wall were 1.59 mm and 1.89 mm. The widths of the inter-radicular septum and the distances from implant to the buccal and lingual plate on different sections were significantly associated with tooth position (*P* < .05).

**Conclusions:**

In the first molar region, the implant is suggested to be placed in the center of the inter-radicular septum, while in the second molar region, the mesial root socket could be considered. Immediate implant placement in the mandibular second molar sockets shows a high risk of IAN injury, lingual perforation, and inadequate primary stability.

## Background

In daily dental practice, the extraction of a mandibular molar is a common phenomenon because of an endodontic failure, caries, or vertical root fracture, and immediate implantation can be considered in this case [1]. Immediate implant placement in molar extraction sites has been reported to be a valid treatment approach with high survival and success rates [2–4]. It has given several clinical benefits, such as reduced surgical procedure, ideal implant positioning, and higher patient satisfaction [4, 5].

Anatomical structures, such as multi-rooted extraction socket, the inter-radicular septum, the inferior alveolar nerve (IAN), and the lingual concavity of submandibular fossa, vary in each patient and pose a challenge in immediate implant placement [6, 7]. The potential surgical complications, for example, inadequate primary stability, IAN injury, and lingual plate perforation may result in the failure of implantation [8, 9]. The choice of implant diameter and length, and an ideal implant insertion position and angle, therefore, are extremely crucial for long-term esthetic and functional success.

Previous studies, evaluating the risk for immediate implant placement in the posterior mandible, have mainly focused on the prevention of lingual plate perforation or IAN injury [6–12]. According to the authors' knowledge, no other paper has tried to discuss the optimal surgical position, and it’s relation to the inter-radicular septum. Furthermore, the horizontal width from the implant surface to the mesial and distal socket wall on different sections was discussed in this study. Moreover, the level where the virtual implant was completely surrounded by bone was identified, offering a more precise measurement of the height of bone available for safe implant placement.

In this study, implants were positioned virtually in a prosthetically driven way in the mandibular first and second molar region. We assessed the relationship between the virtual implant and the related anatomic structures. The purpose of this radiographic study is to explore the optimal immediate implant placement protocol in the mandibular molar region and assess the risk of potential complications.

## Methods

### Patient selection

The study protocol was approved by the Ethics Committee of the First Affiliated Hospital of Zhejiang University (Approval Number: 20191389) and followed the recommendations of the Strengthening the Reporting of Observational studies in Epidemiology (STROBE) guidelines. Cone-beam computed tomography (CBCT) images taken between January 2013 and December 2017 were acquired from the CBCT database of the Department of Stomatology in the First Affiliated Hospital of Zhejiang University. Images were taken by the same radiologist following the manufacturer’s instructions for patient positioning and using the following technical parameters: 110 kV, 2.87 mA, 3.6 s, the full field of view, using the New Tom 3G system (New Tom, QR SRL Company).

The inclusion criteria were as follows: (1) At least 18 years of age; (2) Presence of bilateral permanent mandibular first molar, second molar; (3) Mandibular molars had to be fully erupted and in a normal position; (4) Absence of any anatomic abnormalities or related disease such as periodontitis, which could affect the measurements. CBCT images were excluded if the images were unclear due to artifacts, scattering or other reasons.

### Radiographic measurements

**Fig. 1:**
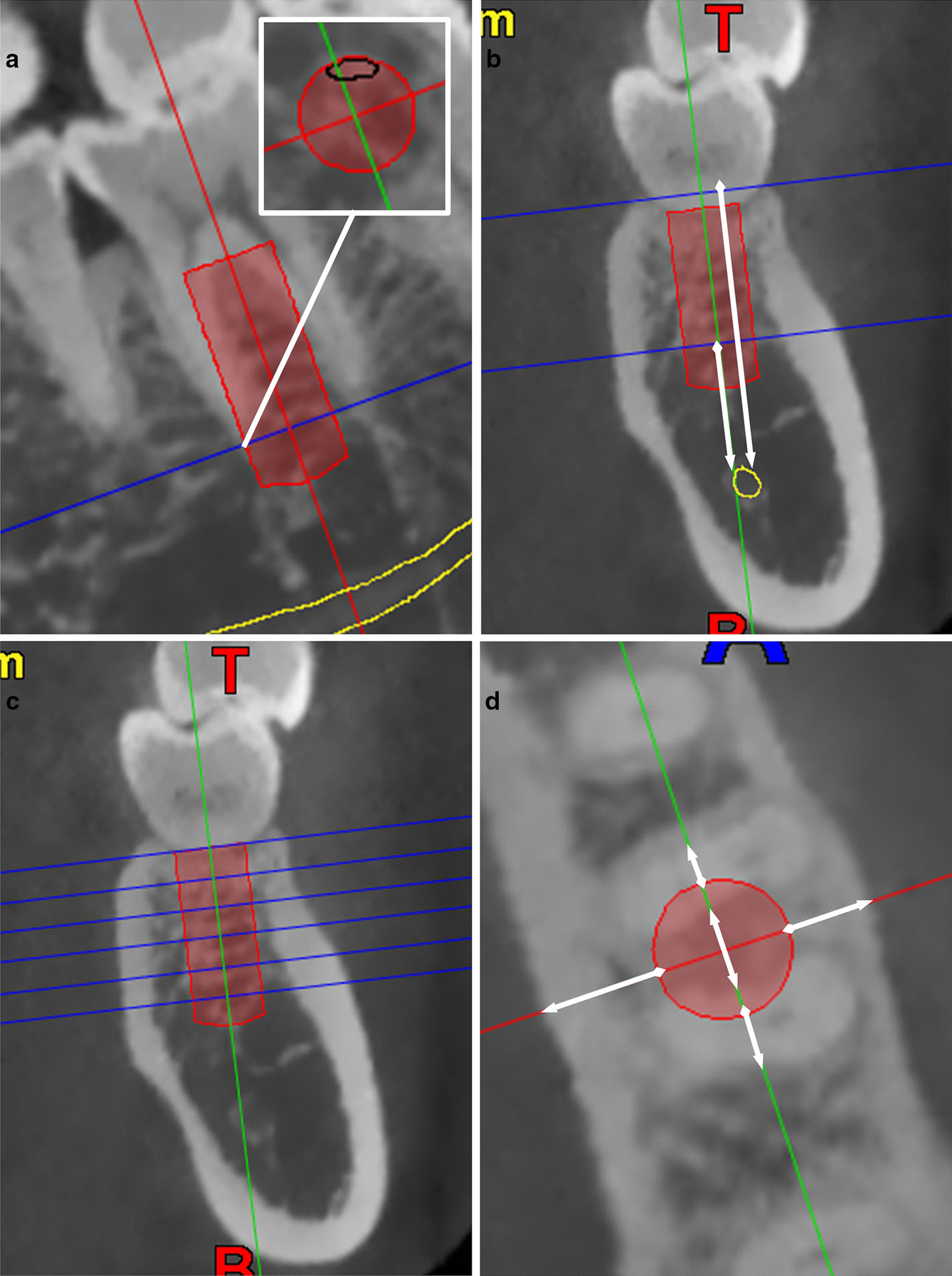
**a** Cross-sectional slice of the investigated molar, virtual implant was placed parallel to the long axis of the tooth (Straumann, bone level regular crossfit, 4.8 × 12 mm), and the level where the virtual implant was completely surrounded was identified. **b** The distance from the alveolar crest to the IAN, and the distance from the level where the virtual implant was completely surrounded by bone to IAN were measured. **c** CBCT sections 1, 3, 5, 7, 9, 11 mm apical to the alveolar crest were obtained. **d** Horizontal widths from the implant surface to the mesial and distal socket wall, the widths of inter-radicular septum, and the distances from implant surface to the buccal and lingual plate in each section were measured

Radiographic measurements were conducted by two examiners, using NNT Viewer software (New Tom, QR SRL Company). The first and second molar regions were investigated in this study. The virtual implants were placed in a prosthetically driven way. The implants were placed based on the position and orientation of the investigated tooth, passing through the central axis of the tooth. The implant platform was placed at 1 mm apical to the alveolar crest (Fig. 1a, b).

Regarding the height of the available bone, the distance from the alveolar crest to the IAN was measured. Furthermore, the level where the virtual implant was completely surrounded by bone was identified, and the vertical distance from this level to the IAN was evaluated (Fig. 1a, b).

The distances from the implant surface to the buccal and lingual plate were measured at the implant platform level. Moreover, the widths of the inter-radicular septum and the distances from implant surface to the buccal and lingual plate were measured at 1, 3, 5, 7, 9, 11 mm apical to the alveolar crest (Fig. 1c, d).

**Fig. 2:**
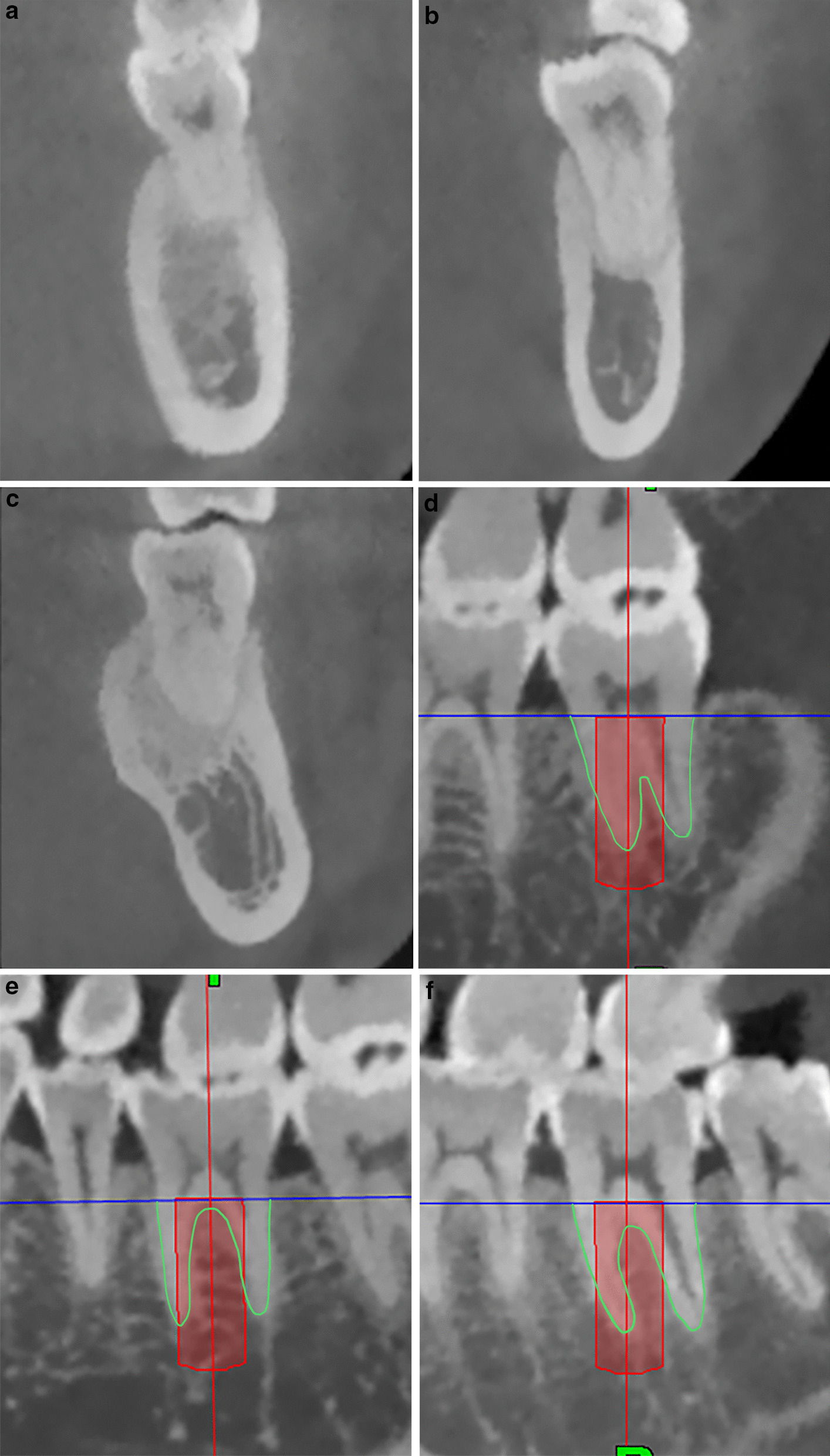
Classification of the cross-sectional morphology of the mandible: **a** convergent type, **b** parallel type, **c** undercut type; classification of the anatomic morphology of the inter-radicular septum: **d** mesial type, **e** septal type, **f** septal–mesial type

The mandibular cross-sectional morphology was categorized into three types, according the classification described by Chan et al. [13]. When the base of the mandibular ridge was wider than its crestal part, the morphology of the mandible was categorized into the convergent (C) ridge type (Fig. 2a). However, when the cross-sectional morphology showed a form of mandible with more or less parallel ridge, it was categorized into the parallel (P) ridge type (Fig. 2b). For the undercut (U) ridge type, the ridge was narrower than its crest, and a lingual undercut on the lingual plate was observed (Fig. 2c). Moreover, the anatomic relationship between the virtual implant and inter-radicular septum was also categorized into three types. When the central axis of the virtual implant only passing through the mesial root, it was categorized into the mesial (M) type (Fig. 2d). When the central axis of the implant only passing through the inter-radicular septum, it was categorized into the septal (S) type (Fig. 2e). For the septal–mesial (S–M) type, the axis passed through both the inter-radicular septum and the mesial root (Fig. 2f). The prevalence of each type for each tooth was calculated.

### Statistical analysis

For the classification of the cross-sectional morphology of the mandible and the inter-radicular septum, a description of frequencies is given. Continuous variables are presented as means and standard deviations (SDs). To determine the normal distribution of the measurements, a Kolmogorov–Smirnov test was performed. Variables were compared using a student’s t-test, or Mann–Whitney U test to identify any significant differences. Moreover, the Pearson correlation was performed to verify the relationship between variations and the patient’s age. The inter- and intra-observer variation was determined by comparing two repeated measurements of the distance from the alveolar crest to the IAN taken 1 month apart using Pearson correlation.

Data processing and statistical analyses were conducted using SPSS (version 25, SPSS Inc). The level of significance was defined as *P* = 0.05.

## Results

Among the 150 subjects included in this study, 75 were females (30.96 ± 6.98 years) and 75 were males (31.68 ± 7.50 years). All sites allowed for a 4.8 mm diameter implant. However, among 600 sites enrolled, perforation of the lingual cortical bone occurred in 12 cases (all occurred in the second molar). The inter- and intra-observer variations were 0.81 and 0.86, respectively.

The distance from the alveolar crest to the IAN was 17.22 ± 2.05 mm in the first molar, and 15.22 ± 1.96 mm in the second molar. The distance from the level where the virtual implant was completely surrounded by bone to IAN was 7.96 ± 2.07 mm in the first molar, and 6.15 ± 2.16 mm in the second molar. Based on the distribution (both *P* < 0.05, Kolmogorov–Smirnov test), regarding both parameters, the differences between the first molar and second molar were statistically significant (both *P* = 0.000, Mann–Whitney U test).

Horizontal widths from the implant surface to the mesial socket wall were 1.47 ± 0.43 mm in the first molar, and 1.70 ± 0.41 mm in the second molar. Horizontal widths from the implant surface to the distal socket wall were 1.94 ± 0.48 mm in the first molar, and 1.84 ± 0.42 mm in the second molar. Based on the distribution (all *P* < 0.05, Kolmogorov–Smirnov test), regarding both parameters, the differences between first molar and second molar were statistically significant (mesial width *P* = 0.000; distal width *P* = 0.007, Mann–Whitney U test). The difference between mesial and distal width in both molar region was also shown to be statistically significant (both *P* = 0.000, Mann–Whitney U test).

The widths of the inter-radicular septum and the distances from the implant surface to the buccal and lingual plate on each section are presented in Table 1. The results demonstrated that all parameters assessed were significantly associated with tooth position (all *P* < 0.05, Mann–Whitney U test).

**Table 1:** The widths of inter-radicular septum and the distances from implant surface to the buccal and lingual plate on each section

Distance to alveolar crest (mm)	First molar			Second molar		
	Septum width	Buccal distance to plate	Lingual distance to plate	Septum width	Buccal distance to plate	Lingual distance to plate
1	0.97 ± 1.09	2.91 ± 0.65	3.46 ± 0.76	0.09 ± 0.43	3.22 ± 0.82	3.98 ± 0.91
3	3.14 ± 0.72	3.07 ± 0.72	4.61 ± 0.88	0.79 ± 0.85	4.45 ± 1.21	4.42 ± 0.80
5	3.82 ± 0.68	3.30 ± 0.86	5.08 ± 0.86	1.48 ± 1.20	5.73 ± 1.46	4.39 ± 0.78
7	4.03 ± 0.86	3.69 ± 1.05	5.17 ± 0.92	1.53 ± 1.33	6.80 ± 1.57	4.27 ± 0.89
9	4.25 ± 1.13	4.03 ± 1.20	5.23 ± 0.96	1.67 ± 1.59	7.44 ± 1.53	3.84 ± 1.01
11	4.66 ± 1.31	4.34 ± 1.33	5.12 ± 1.19	1.92 ± 1.77	7.74 ± 1.64	3.01 ± 1.22

Table 2 demonstrates the distribution of different types of mandibular morphology and the anatomic relationship between the virtual implant and inter-radicular septum.

**Table 2:** Classification of the cross-sectional morphology of the mandible and inter-radicular septum

Tooth	Morphology of the alveolar bone, n (%)			Morphology of the inter-radicular septum, n (%)		
	C	P	U	M	S–M	S
First molar	87 (29.0)	96 (32.0)	117 (39.0)	8 (2.7)	124 (41.3)	168 (56.0)
Second molar	46 (15.3)	51 (17.0)	203 (67.7)	164 (54.7)	105 (35.0)	31 (10.3)

C, convergent; P, parallel; U, undercut; M, mesial; S–M, septal–mesial; S, septal

The relationships between age and the measured parameters resulted in Pearson correlation coefficients varying from − 0.299 to 0.290, showing no, very weak, or weak relationships.

## Discussion

Immediate implant placement of mandibular molars proved to be a viable surgical treatment with a high success rate [3, 14]. The results of this study may be beneficial for surgeons who are considering immediate implantation in the mandibular molar region.

Implant primary stability is the main factor determining the successful osseointegration of dental implants [15]. However, enough primary stability in molar post-extraction sites is often difficult to achieve. Ultra-wide or wide diameter implant increases the engagement and contact area with the inter-radicular septum and socket walls, and therefore, is frequently used in post-extraction molar sites to overcome the lack of primary stability [5, 16–18]. Nonetheless, the long-term prognosis of an ultra-wide diameter implant remains controversial. Some studies reported a higher failure rate of ultra-wide implants than implants of diameter 4 to 6 mm [2]. Recent study demonstrated that the use of implants with < 5 mm diameter is considered to be predictable and successful for immediate implantation in posterior areas [4]. Regular or wide implants also have advantages, such as the low probability of bone dehiscence, lingual perforation, and IAN injury. In this study, therefore, a 4.8 mm diameter virtual implant was used.

An alternative to wide-diameter implants is the placement of implants into the inter-radicular septum [19]. Immediate implant placement into the inter-radicular septum of mandibular molar sockets is a viable treatment option providing favorable outcomes [1, 3, 19, 20]. It was reported that a minimum 3 mm width of the inter-radicular septum was needed to provide initial implant stability [3, 21]. Based on the results presented in Table 1, the implants in the first molar region should be placed 2–3 mm subcrestally to produce better bone-to-implant contact area and obtain adequate primary stability, and less bone resorption may be expected [22]. However, in the second molar region, the inter-radicular septum is often thin or absent, indicating a risk of inadequate primary stability of immediate implant placement.

To identify the most optimal surgical protocol, in this study, we classified the anatomic relationship between the virtual implant and inter-radicular septum into three types: M (mesial), S–M (septal–mesial), and S (septal). The results showed that type S, followed by type S–M, was the most common in the first molar. Therefore, we suggest immediate implant placement in the center of the inter-radicular septum should be considered in the first molar region, which provided favorable outcomes in former studies [1, 3, 19, 20]. However, in the mandibular second molar region, type M had the highest prevalence rate, and the inter-radicular septum is often thin or absent. In this case, the mesial root socket could be considered for immediate implant placement.

Placing an immediate implant in the inter-radicular septum is a complex procedure and that requires a learning curve. During the site preparation, the drill may slip down the slope continually because of the morphology of the inter-radicular septum, leading to the loss of the inter-radicular bone and an inaccurate implant position [21, 23]. Various surgical approaches have been described to place implants in mandibular molar sockets, and using the remaining roots as a guide for implant orientation is the most documented one [1, 20, 21, 24–26]. Implant site preparation guided by the remaining roots ensures predictable ideal implant positioning and orientation [1, 20, 21, 24–26]. Besides, it is crucial to preserve the integrity of the inter-radicular septum and the walls of the extraction socket with an atraumatic tooth extraction [1, 2].

Previous studies have predicted a higher risk of lingual perforation in U type mandible [7]. In this study, a tendency to be classified as U type in more posterior regions was found, which is similar to the results obtained in former studies [7]. Based on the data of the distance between the lingual outer plate surface and the implant surface at the apex in the simulation in this study, the immediate implantation in the mandibular second molar region presents a higher risk of lingual perforation than the first molar region.

To ensure the integrity of IAN, a proper distance between implant and IAN is of utmost importance. Though the results of our study showed that the mean distance from the alveolar crest to the IAN was 16.22 mm, offering sufficient bone height for immediate implant placement. The available bone height in the second molar region was significantly less than the first molar region. This result is similar to previous studies, which indicated that the distance of the IAN is minimal on the distal sections of the second molar [6]. Therefore, to avoid lingual perforation and IAN injury, the maximal implant length in the second molar region should be carefully decided. Besides, the mean distance from the level where the virtual implant was completely surrounded by bone to IAN was 7.06 mm. Considering a safe distance of 1.5–2 mm is recommended between implant and IAN [27], a mean implant anchorage of 5 mm could be obtained in the apical basal bone.

The horizontal gap between the implant and the socket wall is a common finding. In this study, the horizontal widths from the implant surface to the mesial and distal socket wall were 1.59 mm and 1.89 mm. Even though some studies claimed that a large gap (4–5 mm) may heal spontaneously, without the application of regenerative materials [28]. Most authors recommended that augmentation procedures should be considered at the time of implant placement to reduce bone resorption [18, 26].

This study still has some limitations. Firstly, this is a "virtual" study. The inter-radicular septum could be cancellous and of poor bone quality. Placing an immediate implant in a post-extraction molar site is, therefore, a complex and challenging procedure. Moreover, during the measurements, though the inter- and intra-observer variations were high, minor discrepancies may occur in determining the position of the virtual implant. For these reasons, the findings of this study require careful interpretation and should be supported by well-designed, prospective controlled trials with long-term follow-up.

## Conclusions

The present study indicated that immediate implant placement in the mandibular first molar region is a viable treatment. The implant is suggested to be placed into the center of the inter-radicular septum to achieve enough primary stability. However, in the second molar region, the inter-radicular septum is often thin or absent, and the mesial root socket could be considered for immediate implant placement. Besides, the placement of the implant in the mandibular second molar region showed a high risk of IAN injury and lingual perforation. More clinical trials are required to validate these findings.

## Data Availability

The datasets used and/or analysed during the current study are available from the corresponding author on reasonable request.

## Abbreviations

IAN: Inferior alveolar nerve
STROBE: Strengthening the Reporting of Observational studies in Epidemiology
CBCT: Cone-beam computed tomography
